# Mode of Metal Ligation Governs Inhibition of Carboxypeptidase A

**DOI:** 10.3390/ijms252413725

**Published:** 2024-12-23

**Authors:** Jorge Antonio Amador Balderas, Frank Beierlein, Anselm H. C. Horn, Senta Volkenandt, Leon Völcker, Nikoo Mokhtari, Jules Cesar Epee Ndongue, Petra Imhof

**Affiliations:** 1 Computer Chemistry Center, Department for Chemistry and Pharmacy, Friedrich-Alexander University Erlangen Nürnberg (FAU), Nägelsbachstraße 25, 91052 Erlangen, Germany; jorge.ab.amador@fau.de (J.A.A.B.); frank.beierlein@fau.de (F.B.); senta.volkenandt@fau.de (S.V.); leon.voelcker@fau.de (L.V.); nikoo.mokhtari@fau.de (N.M.); jules.cn.epee@fau.de (J.C.E.N.); 2 Erlangen National High Performance Computing Center (NHR@FAU), Friedrich-Alexander University Erlangen Nürnberg (FAU), Martensstraße 1, 91058 Erlangen, Germany; anselm.horn@fau.de; 3 Institute of Biochemistry, Friedrich-Alexander University Erlangen Nürnberg (FAU), Fahrstraße 17, 91054 Erlangen, Germany

**Keywords:** carboxypeptidase A, peptide hydrolysis, zinc coordination, inhibition, molecular dynamics simulations

## Abstract

Carboxypeptidase is a Zn-dependent protease that specifically recognises and hydrolyses peptides with a hydrophobic side chain at the C-terminal residue. According to hydrolysis mechanisms proposed in the literature, catalysis requires a water molecule to be close to the Zn ion so as to be activated as a nucleophile. Among small molecules that resemble the slowly hydrolysed Gly-Tyr peptide, which have been previously designed as inhibitors and characterised structurally, a variant with the terminal amino acid in a D-configuration has been the most effective. Our molecular dynamics simulations of carboxypeptidase complexed with different variants of those inhibitor ligands as well as variants of the Gly-Tyr peptide show that the strength of the inhibitory effect is not related to the binding strength of the ligand. Our data rather support an earlier notion that the inhibition is, at least partially, due to blocking a coordination site at the Zn ion by the ligand coordinating the metal ion in a bidentate fashion.

## 1. Introduction

**Figure 1:**
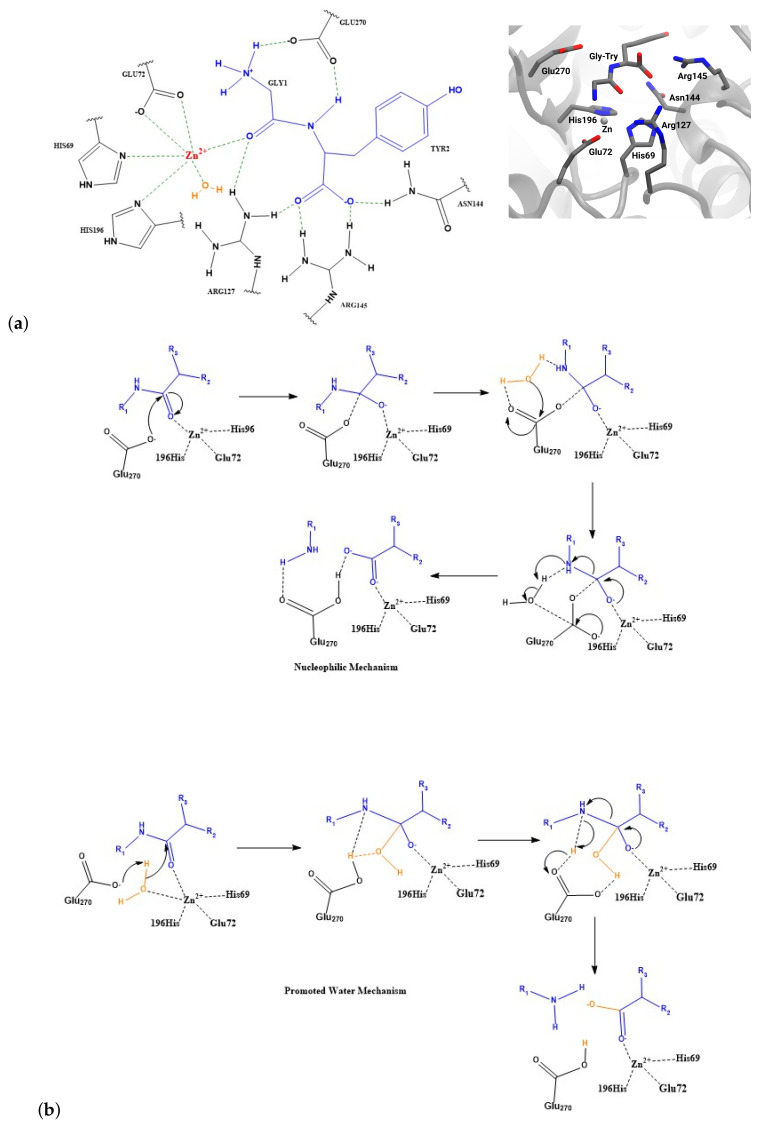
(**a**) The active site of carboxypeptidase A bound to a Gly-Tyr peptide (from crystal structure 3CPA [1]). (**b**) Scheme of the *Nucleophilic Mechanism* and the *Promoted Water Mechanism*. The substrate is highlighted in blue; the attacking water molecule is shown in orange.

Carboxypeptidase A (CPA) is a zinc-containing enzyme that hydrolyses peptides at the C-terminal end and is specific for residues with a hydrophobic side chain. Owing to many solved crystal structures of CPA, with and without a ligand bound [1,2,3], and a number of mechanistic studies [4,5,6], CPA has become a prototypical zinc protease. Among the ligand-bound structures is a small peptide (Gly-Tyr) bound that is only hydrolysed slowly, and its binding mode is hence discussed to reflect a catalytically inactive state [1]. Other solved structures show different small molecule inhibitors, also located close to the central Zn ion. In all those structures, the Zn ion is coordinated by two histidine residues, His69 and His196, one glutamate residue, Glu72, and the ligand in a mono- or bidentate fashion, depending on the ligand. The crystal structure with Gly-Tyr bound [1] further suggests hydrogen bonds between residue Glu270 and the amide nitrogen atom of the ligand, as well as hydrogen bonds between Arg145 and the carboxyl group of the C-terminal tyrosine (see Figure 1).

For the mechanism of the enzymatic peptide hydrolysis, essentially two extremes have been discussed [1,7]: (1.) The first mechanism is a so-called “nucleophilic”, “anhydride”, or “acyl intermediate” pathway in which Glu270 attacks the carbonyl C-atom of the peptide bond and forms an acyl-intermediate that is then hydrolysed by the nucleophilic attack of a water molecule. (2.) The second mechanism is a “promoted-water pathway” or “general-acid-general-base” hydrolysis, which involves direct attack of an activated water molecule, as a hydroxide ion, on the carbonyl C-atom of the peptide bond, formation of a tetrahedral intermediate, and dissociation of the C-terminal residue associated with proton transfer to the nitrogen atom of the departing amino acid (see Figure 1). According to combined quantum mechanical/molecular mechanical calculations [5], the “promoted-water” mechanism is energetically more favourable and hence more probable. This finding is in agreement with ^18^O-substitution experiments that were unable to detect an acyl-intermediate in peptidolysis [8]. Solid state NMR spectroscopy of CPA mixed with Gly-Tyr, on the other hand, revealed a ^13^C signal that the authors assign to an acyl intermediate formed by the attack of Glu270 on the carbonyl C-atom of Gly-Tyr [9].

CPA also hydrolyses esters with similar configurations, and for this reaction, acyl intermediates have been reported, suggesting that proteolysis and esterolysis may follow different pathways [6,10,11]. A computational study, comparing the hydrolysis of a peptide and an ester substrate by CPA, found the formation of an acyl-intermediate from the ester substrate indeed feasible, and therefore its observation possible. The subsequent hydrolysis to the cleaved products, however, is blocked by high barriers. The productive pathway according to that study is a “promoted-water” mechanism for either of the substrates, peptide or ester [6].

The requirement of a “promoted water” molecule, presumably by coordination to the Zn ion and/or proton transfer to the nearby Glu270, appears thus as an emergent consensus. In that context, the lack of a coordination site for such a water molecule was discussed to be a mode of inhibition of CPA. According to the crystal structure, the slowly hydrolysed Gly-Tyr substrate is positioned in the active site, with its carbonyl O-atom coordinated to the Zn ion and the N-terminal N-atom close enough to the Zn ion to represent another coordination partner or at least hinder the binding of a water molecule [1]. Such (almost) bidentate ligation of the central Zn ion was exploited as a design strategy for CPA inhibitors [11,12]. In that work, the authors found that inhibitor variants whose C-terminal moiety has a D-configuration, as opposed to the L-configuration of natural amino acids, were indeed the more effective inhibitors [11,12]. Solving the crystal structures of CPA complexed to some of these inhibitors in their subsequent work [3] supported the notion of the binding mode, namely, a bidentate ligation of the Zn ion, as the governing factor for blocking hydrolysis and hence inhibition of CPA.

**Figure 2:**
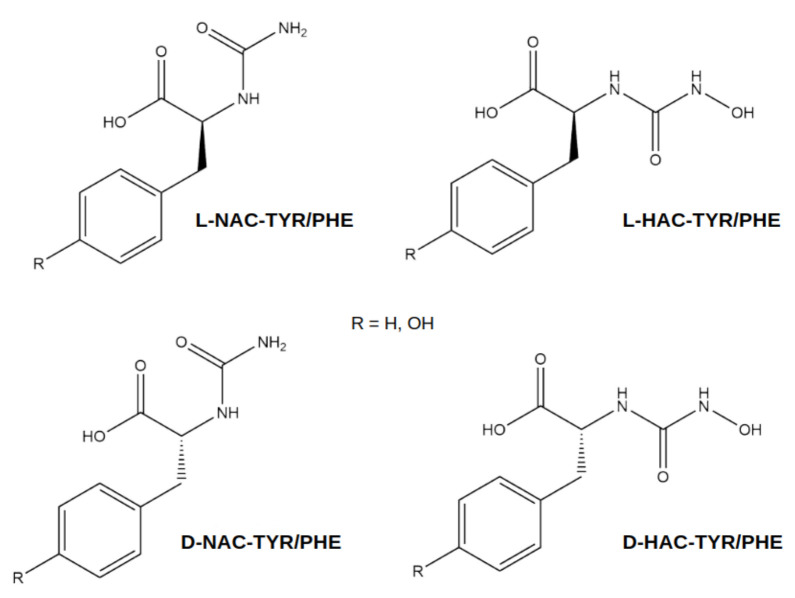
Structures of the quasi-peptide ligands.

In order to obtain insight into the dynamic behaviour, e.g., the conformational flexibility and possible changes in binding poses, of small (quasi-)peptides bound to CPA in solution at finite temperature and to quantify the interactions of the (quasi-)peptide ligands with the enzyme, we performed molecular dynamics simulations of CPA–ligand complexes. The ligands are inhibitor molecules, containing a hydroxamate moiety, as designed in ref. [11] (cf Figure 2), and the slowly hydrolysing Gly-Tyr substrate that putatively is also an inhibitor rather than a true substrate. For a fair comparison between the different ligands, we also modelled Gly-Tyr in a variant with neutral N-terminus, as is the case in the quasi-peptide ligands, and the latter were additionally modelled in a variant with a tyrosine at the C-terminus, like in Gly-Tyr, rather than the phenylalanine residue in the designed inhibitor molecules.

## 2. Results

**Figure 3:**
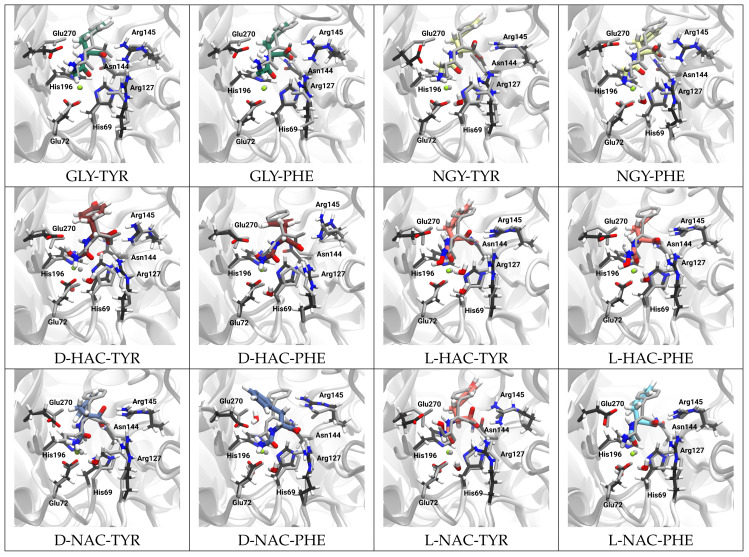
Medoid structures of CPA bound to the different ligands (coloured), overlaid with the respective template crystal structure (grey) listed in Table 1.

Figure 3 shows snapshots (medoid structures) of the simulated systems, overlaid with the crystal structure from which the models were derived (see also Table 1).

In all models, the ligand remained well placed in the active site, which can partly be attributed to the explicitly modelled bond to the Zn ion. Likewise, all other residues coordinating the Zn ion remained in place, except for a somewhat displaced side chain of Arg145 in some of the systems (NGY-PHE, D-NAC-PHE). The side chain of Glu270 is slightly rotated with respect to the crystal structure in at least one simulation run in all of the systems. In the model with D-NAC-TYR or D-NAC-PHE bound to CPA, the tyrosine or phenylalanine, respectively, of the ligand deviate in their conformation from the crystal structure. This is due to the ligands being different stereo-isomers, i.e., with D-Tyr or D-Phe, respectively, in the model and L-Tyr or L-Phe, respectively, in the ligand of the crystal structure.

### 2.1. Fluctuations

**Figure 4:**
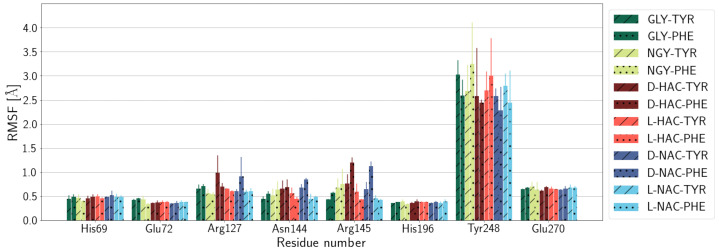
Fluctuations of selected active-site residues of the protein. The fluctuations of the all protein residues are shown in Figure S1. Note that His69, Glu72, and His196 are bound to the Zn ion by harmonic potentials (see Section 4). Different colours refer to different N-terminal parts of the ligands (see legend). C-terminal tyrosine and phenylalanine are indicated by diagonal lines or dots, respectively. Error estimates are calculated from the standard deviation from the mean of the three simulation runs (n=3) per system.

Protein residues of the active site that are ligated to the Zn ion, i.e., His69, Glu72, and His196, fluctuate only very little in all models (see Figure 4). Glu270 fluctuates more than the protein residues that are close to the Zn ion but by a similar amount in all models. Marginally larger fluctuations are observed for residues Arg127, Asn144, and Arg145 in some of the models. We have also analysed the fluctuations of Tyr248, which are significantly larger than those of the protein residues closer to the ligand and also have a larger error. The flexibility of this residues is, however, comparable in all models. Of note, it is also similar in models with Tyr or Phe as the C-terminal part of the ligand, indicating that there is no particular interaction of Tyr248 with the hydroxyl group of the ligand’s Tyr residue, if present. Also, within error, the distances between Tyr248 and the ligands are comparable in all models (see Figure S5).

**Figure 5:**
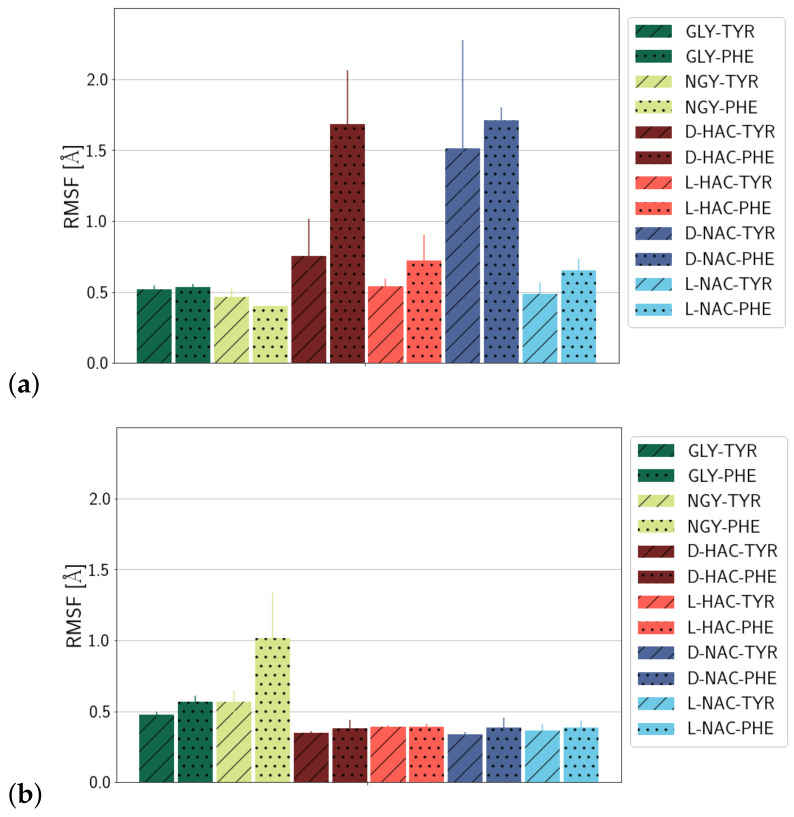
Fluctuations of the ligand (**a**) N-terminal part and (**b**) C-terminal part. Different colours refer to different N-terminal parts of the ligands (see legend). C-terminal tyrosine and phenylalanine are indicated by diagonal lines or dots, respectively. Error estimates are calculated from the standard deviation from the mean of the three simulation runs (n=3) per system.

According to the fluctuations computed for bound ligands (see Figure 5), the C-terminal parts of all ligands fluctuate very little (∼0.5 Å) and by a similar amount, suggesting that the C-terminal end is well accommodated in the active site of the protein. The N-terminal part of most of the ligands fluctuates similarly little (∼0.5 Å). However, for two models with D-forms, D-HAC-PHE and D-NAC-PHE (and for D-NAC-TYR, but with large error), higher fluctuations of the N-terminal parts are observed than in the other models. This indicates a less optimal fitting of the ligand into the active site of the protein, possibly enhanced by fewer or weaker interactions of the active site to the Phe rings compared to those with a Tyr residue.

### 2.2. Zn Ion Coordination

**Figure 6:**
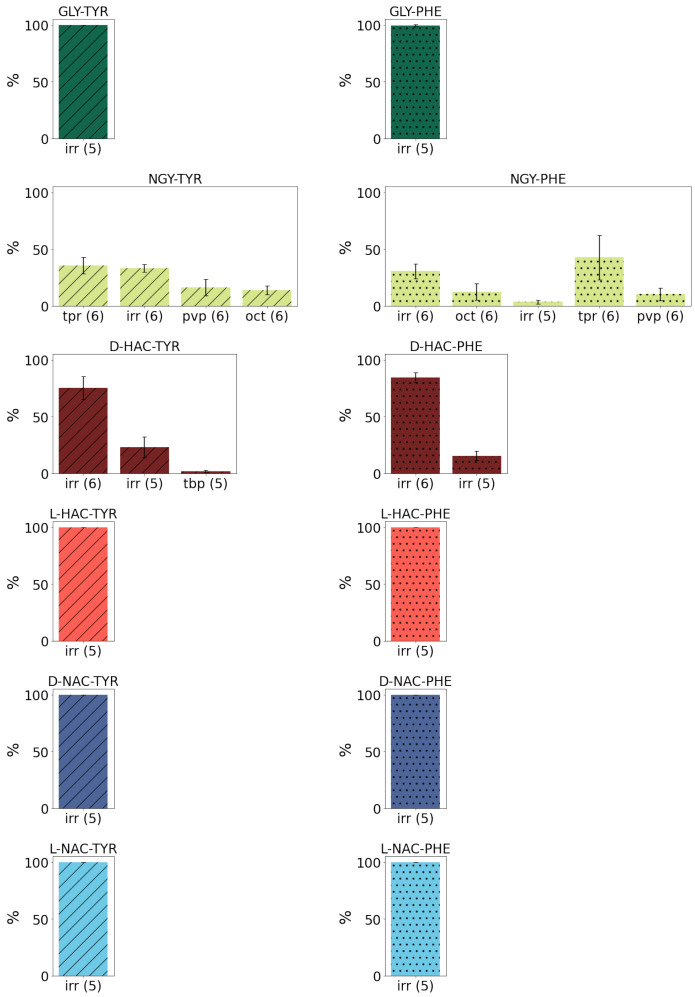
Coordination geometry of the Zn ion (irr: irregular; trp: trigonal prism; oct: octahedron; pvp: pentagonal bipyramid; tbp: trigonal bipyramide). Different colours refer to different N-terminal parts of the ligands. C-terminal tyrosine and phenylalanine are indicated by diagonal lines or dots, respectively. Error estimates are calculated from the standard deviation from the mean of the three simulation runs (n=3) per system.

The Zn ion coordination in most models is irregular, with a coordination number of 5. One notable exception are the models with the D-HAC-TYR/PHE ligands that show predominantly coordination number 6 but also in irregular geometry. The only model which exhibits regular geometries, in addition to irregular ones, are NGY-TYR and NGY-PHE, for which trigonal bipyramids (tbp) can be observed, albeit in only 35% and 40%, respectively, of the simulation frames (see Figure 6).

**Figure 7:**
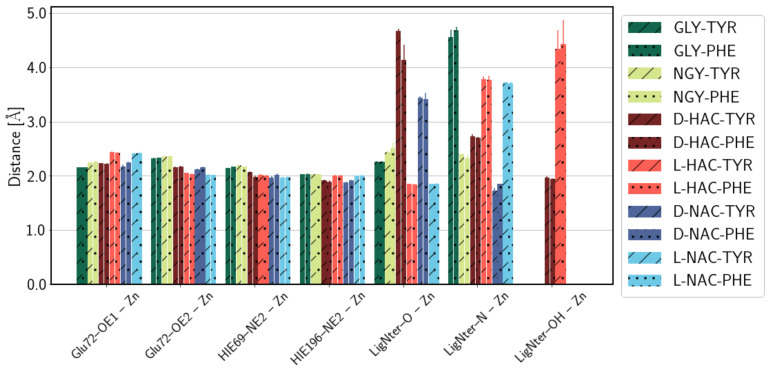
Distances between the Zn ion and protein residues of the active site. Different colours refer to different N-terminal parts of the ligands (see legend). C-terminal tyrosine and phenylalanine are indicated by diagonal lines or dots, respectively. Error estimates are calculated from the standard deviation from the mean of the three simulation runs (n=3) per system.

From the analysis of the distances between coordinating residues and the Zn ion (see Figure 7), it becomes clear that the two histidine residues and Glu72 as a bidentate ligand form a stable coordination sphere and remain close to the pre-assigned values in the bound EZAFF model for Zn ions. The fifth coordination site is occupied by the ligand, albeit with different atoms, depending on the ligand: GLY-TYR/PHE, NGY-TYR/PHE, L-HAC-TYR/PHE, and L-NAC-TYR/PHE coordinate the Zn ion with their carbonyl oxygen atom; D-NAC-TYR/PHE coordinate the Zn ion with their terminal amino N-atom (which occupies a sixth coordination site in the models of the NGY-TYR/PHE ligands); and D-HAC-TYR/PHE has its hydroxyl O-atom close to the Zn ion (see Figure 7). The variation in Zn-coordinating atoms between ligands in D- or L- configuration can be explained by the (slightly) different orientation of those ligands. D-HAC-TYR/PHE, moreover, deviate even further by using yet another atom. The distance between D-HAC’s terminal N-atom to the Zn ion is, however, short enough (<2.8 Å) to qualify for a coordinative interaction with the Zn ion, giving rise to the sixfold coordination with irregular geometry.

### 2.3. Interaction Energies

**Figure 8:**
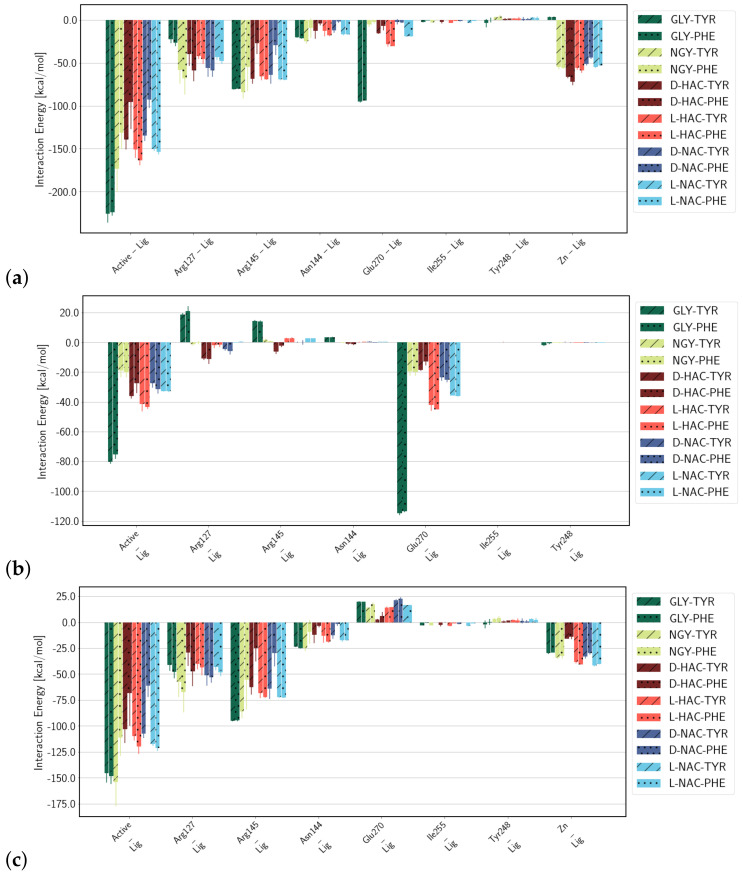
Electrostatic interactions between active-site residues and (**a**) the entire ligand, (**b**) the N-terminal, and (**c**) the C-terminal part of the ligand. Error estimates are calculated from the standard deviation from the mean of the three simulation runs (n=3) per system.

**Figure 9:**
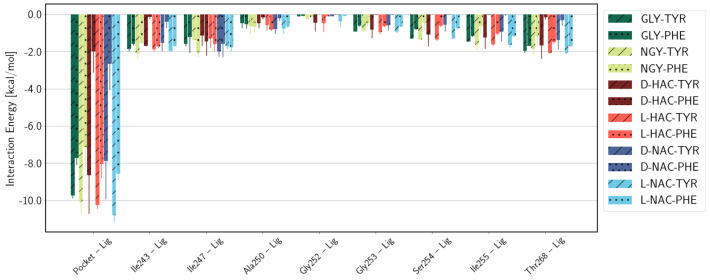
Van der Waals interactions between residues of the hydrophobic pocket and the C-terminal part of the ligand. Different colours refer to different N-terminal parts of the ligands (see legend). C-terminal tyrosine and phenylalanine are indicated by diagonal lines or dots, respectively. Error estimates are calculated from the standard deviation from the mean of the three simulation runs (*n* = 3) per system.

As anticipated, the electrostatic interactions of the ligand with residues of the active site are governed by the charge of the respective part of the ligand, i.e., positive or neutral N-terminal or negative C-terminal part, and the charge of the active-site residue in question (Figure 8). The N-terminal part of the ligand interacts significantly only with the two arginine residues Arg127 and Arg145, and with Glu270. Regarding the former, the GLY-TYR and GLY-PHE ligands that carry a positive charge at the N-terminus interact unfavourably with the positively charged Arg residues, despite the latter being located on the C-terminal side, and strongly favourably with the negatively charged Glu270. Also, the other ligands exhibit a favourable interaction of their N-terminal part with Glu270, the strength of which depends on the stereo-isomer, that is, L-forms interact more strongly than the respective D-forms. It is interesting to note that the electrostatic interactions of the C-terminal part of the ligand with active-site residues differ to some extent as well. Some of the variability is between ligands with C-terminal ends being Tyr or Phe and, hence, anticipated due to the difference in composition. But there are also differences between the amino acid ligands, GLY-TYR/PHE and NGY-TYR/PHE, and the other ligands in such that the peptide ligands interact more strongly with Arg145 and Asn144. This may be due to the quasi-peptide ligands lacking a Cα atom and thus being shorter than the peptide ligands. They may therefore not fully span from the Zn ion as a binding site for the N-terminus to the binding site for the carboxyl terminus. Moreover, the D-form ligands with Phe as the C-terminal residue exhibit significantly lower interactions with Arg145 and Asn144, respectively, than their counterparts with Tyr as the C-terminal residue. This is due to larger distances between these protein active-site residues and the D-HAC-PHE and D-NAC-PHE ligands (see Figure S3). Those and their considerable fluctuation (judged from the error estimates) render the D-Phe less well-positioned than D-Tyr or either of the L-forms. This is further supported by the differences in van der Waals interactions between the protein residues forming the hydrophobic pocket (according to refs. [13,14]) and the C-terminal part of the ligands: ligands with Tyr as the C-terminal residue exhibit generally stronger interactions with the hydrophobic pocket than ligands with Phe as the C-terminal residue. For the D-forms D-HAC-TYR/PHE and D-NAC-TYR/PHE, this difference is particularly pronounced (see Figure 9).

The van der Waals interactions of the ligands with protein residues of the active site (see Figure S2) are significantly weaker than the corresponding electrostatic interactions, except for interactions with the unpolar Ile255 and, of note, also Tyr248.

Judging from the total of interaction energies, ligands D-HAC-PHE and D-NAC-PHE are least strongly bound. GLY-TYR/PHE are the most strongly bound ligands, owing to the attraction of the opposite charges of the N-terminus and Glu270. For the same reason, i.e., the positive charge at the N-terminus, this ligand experiences no further attraction by the Zn ion, in contrast to the other ligands.

### 2.4. Hydrogen Bonds

**Figure 10:**
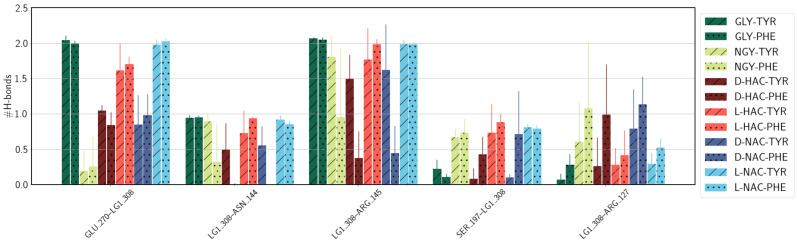
Hydrogen bonds between active-site residues and the ligand. For details of the donor and acceptor atoms, see Supplementary Figure S12. Different colours refer to different ligands (see legend). C-terminal tyrosine and phenylalanine are indicated by diagonal lines or dots, respectively. Error estimates are calculated from the standard deviation from the mean of the three simulation runs (n=3) per system.

The ligands are held in the active site of the carboxypeptidase A protein not only by coordination to the Zn ion, but also held by hydrogen bonds to protein residues of the active site. These are up to two hydrogen bonds with Glu270 for the models GLY-TYR/PHE and the L-forms of HAC and NAC ligands, and one for the other ligands, except for NGY-TYR/PHE (see Figure 10). Also, Arg145 forms on average more than one hydrogen bond with ligands containing Tyr but not with those that have a Phe at their C-terminus (except for GLY-PHE). Asn144 is hydrogen-bonded to all but the D-forms of the PHE ligands. For Ser197, in contrast, the hydrogen bond probability to the D-HAC-PHE and D-NAC-PHE is the lowest. These two residues, however, exhibit hydrogen bonds to the amide group of NGY-TYR/PHE and ligands L-HAC-PHE and L-NAC-PHE, indicating that it is the placement of the ligand that allows this interaction or not.

For the L forms of the HAC and NAC ligands, a hydrogen bond between Asn144 and Arg145 is observed that has significantly lower probability in the other models (see Figure S13).

### 2.5. Water Distribution

**Figure 11:**
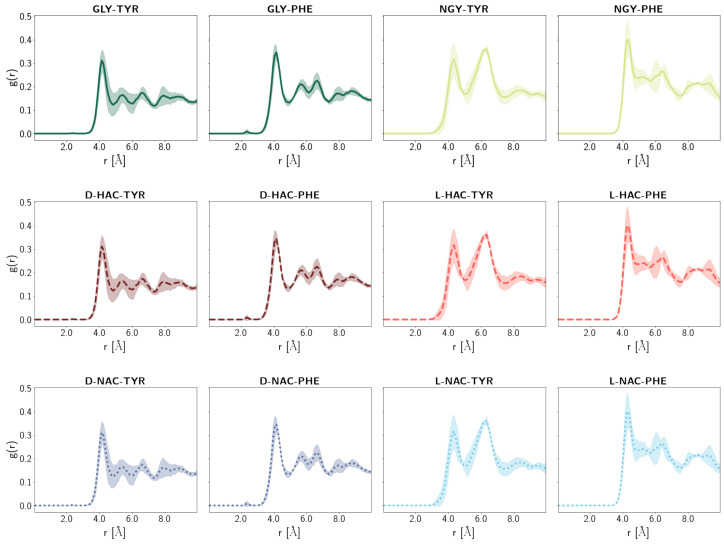
Radial distribution function of water molecules around the Zn ion. Different colours refer to different ligands. Different N-terminal parts are indicated by different line styles. Error estimates are calculated from the standard deviation from the mean of the three simulation runs (n=3) per system.

All models show a radial distribution function of water molecules around the Zn ion with a peak at ∼4Å, which is not quite within coordination distance (see Figure 11). The L-forms of the HAC-TYR and NAC-TYR ligands and the NGY-TYR ligand exhibit a second strong peak at ∼6Å that is also present in the corresponding ligands with Phe instead of Tyr, but significantly less pronounced. The D-forms of those ligands and the charged amino acid ligands GLY-TYR/PHE all have two peaks in that region, one at ∼5.5Å and the other one at ∼6.5Å, indicating two water positions in that distance.

**Figure 12:**
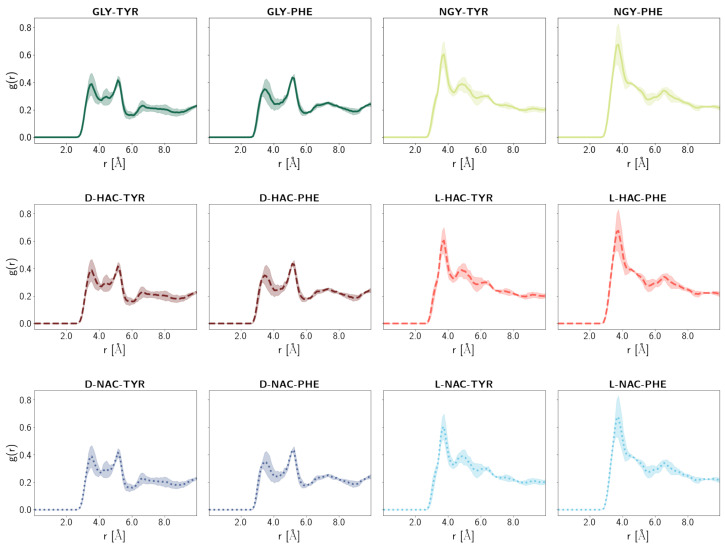
Radial distribution function of water molecules around the carbonyl C-atom of the ligand. Different colours refer to different ligands. Different N-terminal parts are indicated by different line styles. Error estimates are calculated from the standard deviation from the mean of the three simulation runs (n=3) per system.

Also, the radial distribution function of water molecules around the carbonyl C-atom of the ligands (Figure 12) reveals a pattern within the ligands: The L-forms of the HAC-TYR and NAC-TYR ligands and the NGY-TYR ligand exhibit a strong peak at ∼3Å and a smaller one at ∼5Å. The corresponding D-forms and the GLY-TYR/PHE ligands, in contrast, show two smaller peaks of comparable height at about the same positions. At least the peak at the shorter distance, the one that is featured more strongly in the L-forms, is indicative of a water molecule placed in a distance suitable for nucleophilic attack on the carbonyl C-atom of the ligand.

## 3. Discussion

According to [3], D-HAC-PHE has been identified as the best inhibitor among the quasi-peptide ligands D-HAC-PHE, L-HAC-PHE, and L-NAC-PHE. Our MD simulation data suggest that the stronger inhibitory effect, compared to the other two quasi-peptide ligands, cannot be attributed to a stronger binding affinity. Computed interaction energies and the average number of hydrogen bonds are less favourable than, or at best comparable to, those values computed for the other ligands.

Of note, also, the D-NAC-PHE ligand, for which no crystal structure is available and no inhibitory effect was observed [3], shows weaker interactions with the active site of CPA than its corresponding L-isomer. In this case, the weak interactions of D-NAC-PHE with CPA may indeed result in too low of a binding affinity of this ligand to be trapped in a crystal structure and/or act as an inhibitor. One has to keep in mind that the bonded Zn-model, used in the present MD simulations, ties the ligand to the active site, and a dissociation of an unfavourably bound ligand from the protein could simply not be observed.

The D-form ligands D-HAC-PHE and D-NAC-PHE exhibit the largest distances to the arginine residues, Arg127 and Arg145, that are supposed to recognise and stabilise the binding of a carboxy-terminus. This, together with the larger fluctuations of these D-form ligands, further suggests that the (stronger) inhibitory effect of D-HAC-PHE cannot be explained by this ligand binding particularly well to the active site of CPA.

The Tyr variants D-HAC-TYR and D-NAC-TYR exhibit interactions (energies, hydrogen bond, and distances) with CPA’s active site that are comparable to those of the respective L-forms and at least the peptide ligands NGY-TYR/PHE with neutral N-termini, suggesting that their binding affinities are comparable. Only the lower hydrogen-bond probabilities with Glu270 render the D-forms D-HAC-TYR and D-NAC-TYR less favourably bound than their respective L-isomers.

One can argue, though, that a reasonably but not perfectly bound ligand that cannot be processed by the enzyme constitutes a better inhibitor than a well-bound ligand that, eventually, undergoes hydrolysis. According to our data, in all models, there are water molecules close to the Zn ion and, perhaps more importantly, close enough to the carbonyl C-atom of the ligand to allow nucleophilic attack. The probability of a water molecule in such a near-attack distance is, however, significantly higher for the L-forms of the quasi-peptide ligands, and the NGY-TYR/PHE peptide with neutral glycine, than in the D-forms or the charged GLY-TYR/PHE peptide ligands. These data may suggest that the approach of a water molecule is somewhat hindered by the configuration of the D-form ligands.

**Figure 13:**
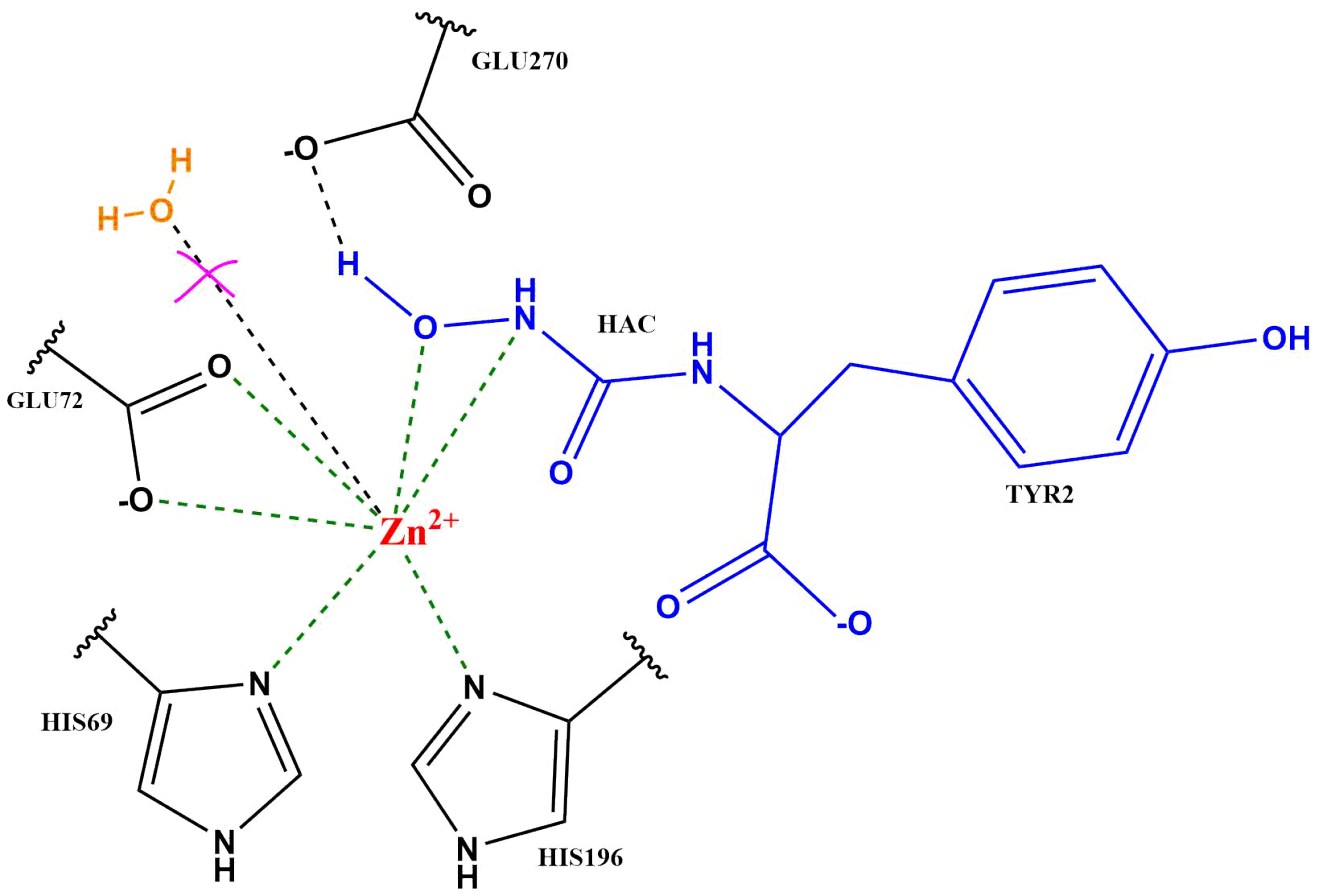
Ligands D-HAC/TYR-PHE (in blue, only Tyr is shown) coordinate the Zn ion (in red) in a bidentate fashion, thereby blocking the coordination of a water molecule (orange) and hence its activation as a nucleophile.

In all systems, the coordination geometry of the Zn ion remains irregularly fivefold, with the exception of the D-HAC-TYR/PHE ligand and the NGY-TYR/PHE models, in which a sixfold coordination is observed. In the latter, this is due to the nitrogen atom of the neutral N-terminus not being hydrogen-bonded to Glu270, as is the case in its charged counterpart, GLY-TYR/PHE, but moving towards the Zn ion. In the D-HAC-TYR/PHE models, the OH group and the terminal N-atom form the two coordination partners for the Zn ion. The fivefold coordination, initially enforced by the bonded Zn model, is hence flexible enough to allow an increase in coordination number, at least by a bidentate ligand. Such a behaviour is, interestingly, not observed for any of the other quasi-peptide ligands, which could also coordinate the Zn ion with the carbonyl oxygen atom and the nitrogen atom of the N-terminus. Instead, these ligands use only one of the two atoms, depending on the D- (which use the N-atom) or L-form (which ligate by the O-atom). Such a non-optimal placement of the ligand and binding to the central Zn ion may, however, allow a water molecule to enter the coordination sphere of the Zn ion and thus become activated as a nucleophile. In contrast, with six coordination sites occupied by protein residues or the ligand, coordination by a water molecule, and hence its activation and subsequent attack as a nucleophile, becomes less probable (see Figure 13 for a schematic illustration). The bidentate ligation was also discussed earlier, based on the crystal structure of CPA bound to the inhibitor ligands [3], as the mode of inhibition.

These arguments render the NGY-TYR/PHE ligands with neutral glycine also inhibitors of CPA. While that may seem counter-intuitive, since a neutral amino group is more similar to the amide group in a longer peptide chain, the actual substrate of CPA, the pyramidal configuration of the amino N-atom, is likely better suited to coordinate the Zn ion than that of an N-atom in a peptide bond. We do not find a bidentate coordination of the Zn ion in the simulations of models with charged glycine, which somewhat contrasts the coordination in the crystal structure and is the rationale for Gly-Tyr being hydrolysed only slowly by CPA [1]. One can therefore argue that the crystal structure more likely represents NGY-TYR rather than GLY-TYR, which is supported by the pH of 7.4 at which the crystals were prepared [1]. Both our models and the crystal structure, however, suggest a hydrogen between Glu270 and the N-terminus of the Gly-Tyr peptide with a charged N-terminus. In this light, it may also be conceivable that a proton transfer from the NH3 group of Gly to Glu270 has occurred in the experimental structure, a process that cannot be observed in our classical MD simulations. Proton transfer from the activated water molecule to Glu270, and subsequent nucleophilic attack of the thus-formed hydroxide ion, is indeed part of the hydrolysis mechanism, according to calculations in truncated enzyme models [4] as well as in QM/MM studies [5]. With a proton already accepted from the N-terminus of the ligand, this route for formation of the nucleophile is blocked, even if a water molecule could coordinate the Zn ion, and thus another rationale for the Gly-Tyr peptide with a charged N-terminus being processed only poorly by CPA.

Structures of inhibitors consisting of a phosphonic ester group [2] suggest that the active site of CPA is actually much better tuned to accommodate a tetrahedral intermediate (or transition state) rather than a substrate with a planar peptide bond. Such an idea would also explain the tolerance for CPA to bind D- and L-forms of the C-terminal amino acid, that is, no perfect match or lock and key is required for substrate binding as long as the carboxyl group of the C-terminus is facing the arginine residues (mainly Arg145). The hydrophobic side chain is accommodated in the hydrophobic pocket at the expense of the second but last residue being imperfectly placed, but a tolerable one as long as the Zn ion is still somehow coordinated by the ligand.

An inhibitory effect (of D-HAC-PHE) that does not rule out the processing of the NGY-TYR/PHE ligands by the CPA enzyme could therefore be by a trapping of a tetrahedral acyl intermediate after the attack of Glu270 on the central carbonyl C-atom of the (quasi-)peptide bond. Such a hydrolysis mechanism with the formation of an acyl intermediate has been found unlikely for peptide substrates, but at least the transient formation of an acyl intermediate cannot be ruled out for carboxyl esters [6,8,10,11,15]. Hydrolysis of (hydroxy-)aminocarbonyl-phenylalanine or -tyrosine may, however, be a borderline case of peptidolysis, following a slightly different mechanism. Judging from the distances between the closest carboxyl oxygen atom of Glu270 to the central carbonyl atom as the site of attack (see Figure S14), all ligands stand a chance of becoming attacked by Glu270, except for perhaps NGY-TYR/PHE, for which this distance is somewhat larger than in the other models (and which as a true peptide would rather follow a “promoted water” mechanism). Of the quasi-peptide ligands, the L-forms exhibit slightly shorter Glu270-O–ligand-C distances than the D-forms and have a larger probability of hydrogen bonds of the ligand with Glu270, which can be considered as keeping this residue closer and, hence, more probable to attack. In the D-HAC-TYR/PHE models, Glu270 is furthermore hydrogen-bonded to the N-terminal OH group of the ligand, effectively reducing this residue’s negative charge and thereby its nucleophilicity. Whereas the formation of an acyl intermediate may thus be less likely in the (presumably) better inhibitors, D-HAC-TYR/PHE, the probability for such an intermediate to be trapped may still be larger, rationalised by the same arguments as “direct” hydrolysis through a promoted water mechanism: The further processing of the acyl intermediate to the cleaved product also requires the attack of a water molecule. In the L-form ligands L-HAC-TYR/PHE and L-NAC-TYR/PHE, and in D-NAC-TYR/PHE, with a fivefold coordination of the Zn ion, there is a free binding site for such a water molecule, while in D-HAC-TYR/PHE there is not. Moreover, the probability for a, non-neccessarily Zn-coordinated, water molecule to be close to the carboxyl C-atom of Glu270 is lower in the D-forms (and GLY-TYR/PHE) than in their L-form counterparts. It is therefore also conceivable that the combination of a lower probablity of the formation of a tetrahedral acyl intermediate in D-HAC-TYR/PHE than in the other systems with quasi-peptide ligands, together with the presumably longer lifetime of such an intermediate, result in the strongest inhibitory effect.

Carboxypeptidase B, a carboxypeptidase that cleaves the C-terminal amino acid, specific for basic side chains, is also a zinc-dependent carboxypeptidase. According to available crystal structures, the zinc-coordination, at least without an inhibitor or substrate bound, is similar to that in carboxypeptidase A: two histidine residues and one glutamate residue are in bonding distance to the zinc ion (1Z5R [16]). A fourth coordination site is occupied by, e.g., the sulphur atom (1ZG7, 1ZG8, 1ZG9 [16]), selene atom [17], or a phosphate oxygen atom (2PIY, 2PIZ, etc. [18]) of an inhibitor. Note that the zinc-coordinating glutamate is a mono-dentate ligand in carboxypeptidase B, whereas it is a bidentate ligand in carboxypeptidase A, also with (quasi-)peptide ligands or phosphonate inhibitors [2] bound. Another glutamate residue is positioned to play a role comparable to that of Glu270 in carboxypeptidase A, and nearby water molecules, though not coordinating the zinc ion, which may act as a nucleophile. One could therefore envisage a similar hydrolysis mechanism for the two enzymes. Inhibition by an additional coordination of the zinc ion may, however, in carboxypeptidase B be less efficient than in carboxypeptidase A since this would lead to only a fivefold coordination, compared to the fourfold coordination suggested by the crystal structures, hence still leave a binding site for a water molecule and hence the possibility for its activation and subsequent nucleophilic attack. On the other hand, the second oxygen atom of the coordinating glutamate residue is close enough to the zinc ion to allow its coordination after small rearrangements, an adjustment that may well occur upon the binding of a (quasi-)peptide ligand. Further studies are necessary to explore this scenario.

For other zinc-dependent peptidases, our findings on the inhibition of the hydrolysis reaction by blocking the coordination sites at the zinc ion may be transferable. Such an inhibition mechanism, however, is subject to the putative nucleophilic water molecule occupying the sixth, and hence “last” coordination site at the zinc ion. This mechanism does not depend on the enzyme’s preference for the N-terminus (in aminopeptidases) or the C-terminus (in carboxypeptidases) or the nature of the amino acid at the cleavage site, as long as the (pseudo-)substrate has the scissile peptide bond positioned close to the zinc ion.

## 4. Materials and Methods

### 4.1. Model Setup

We have built models for carboxypeptidase A with the following ligands bound: a Gly-Tyr peptide with a charged N-terminus and C-terminus, the same peptide but with a neutral N-terminus, D- and L-[(amino)carbonyl]phenylalanine, and D- and L- (N-hydroxyamino)carbonyl]phenylalanine. In addition, we modelled all ligands with the tyrosine replaced by a phenylalanine and vice versa. All models are based on PDB structures with the same or similar (e.g., a phenylalanine instead of a tyrosine) ligands in the active site of CPA. The D-variants of [(amino)carbonyl]tyrosine and [(amino)carbonyl]phenylalanine were modelled based on the structure of D-(N-hydroxyamino)carbonyl]phenylalanine. The list of the models, by names for the ligands used in this work, and the PDB codes of the crystal structures used to build the models can be found in Table 1.

### 4.2. Parameterisation

Parameters for the N-terminal protein residues NNG (N-terminal neutral glycine), NAC (N-terminal N-aminocarbonyl), and HAC (N-terminal N-hydroxyaminocarbonyl) for ff14SB [19] were obtained as follows:

The (quasi-)peptide ligands were extracted from PDB [20,21] structures 3CPA [1], 1HEE [3], and 1HDU [3]. All three N-terminal residues were capped with N-methyl amine, and missing hydrogen atoms were added with Avogadro [22]. Since these terminal residues are small and achiral (the D and L-form of the ligands refers to the C-terminal tyrosine or phenylalanine, respectively), only a single conformation was used for atomic charge generation.

The capped ligand systems were optimised with Gaussian 16 [23] on the HF/6-31G(d) level; refs. [24,25,26,27,28,29,30,31,32,33] frequency calculations ensured the true minimum character of the conformations found. Atomic charges were obtained using the RESP [34,35] approach via the R.E.D. server [36,37]. Atom types were assigned according to ff14SB, so that most force field parameters were taken from that force field, and missing parameters were amended from gaff2 [38,39].

Due to its general approach, our parameterisation allows for a straightforward generation of different ligand systems using the new N-terminal residues as building blocks. Parameter files for NGY, NAC, and HAC are available in the Supplementary Material.

Bonds and charges between the zinc ion and its surrounding ligands were parameterised using the metal center parameter builder MCPB.py [40,41]. This involved generating a large model comprising the ion and its nearby ligands. Hydrogen atom positions in this model were optimised using Gaussian 16 [23] with the B3LYP functional and the 6-31G(d) basis set [24,25,26,27,28,29,30,31,32,33,42]. Partial charges were subsequently calculated via the RESP fitting procedure with the ChgModB scheme [43], ensuring the charges of backbone heavy atoms matched AMBER ff14SB values [19]. Bond and angle force constants were derived based on an empirical relationship between equilibrium bond/angle values and force constants.

### 4.3. Molecular Dynamics Simulations

The systems were solvated with TIP3P [44] water in a truncated octahedral box, extending 15 Å beyond the solutes in all directions. Sodium counter-ions were introduced to neutralise the system [45], with additional NaCl added to achieve a salt concentration of 150 mM.

The protein was treated by the ff14SB force field [19], while the ligands were described by gaff2 parameters, with RESP charges obtained as described above. The zinc ions were treated with the bonded Extended Zinc AMBER Force Field (EZAFF) model [46], which has proven to well-preserve the geometries observed in crystal structures [47].

After initial geometry optimisation (first 5000 steps with restraints (50 kcal mol^−1^ Å^−2^) on the ligands, then 5000 optimisation steps without restraints (switch from the steepest descent to conjugate gradients after 500 steps in either case), the solvated systems were heated to 310 K during a 500 ps simulation, with weak restraints (10 kcal mol^−1^ Å^−2^) on the ligands in the NVT ensemble. After that, 1000 ns unrestrained NPT Langevin dynamics with a time step of 2 fs were performed for each simulation system at 310 K and 1 bar (weak pressure coupling, isotropic position scaling, pressure relaxation time 2 ps, collision frequency 2 ps^−1^). SHAKE constraints were applied to bonds involving hydrogen [48]. Periodic boundary conditions were used throughout, and the distance cutoff for all non-bonding interactions was set to 10 Å. Long-range electrostatics were described by the particle-mesh Ewald method [49,50]. For van der Waals interactions beyond those included in the direct sum, a continuum model correction for energy and pressure was used, as implemented in Amber. Each system was simulated in three replicas (n=3); coordinates were saved every 100 ps.

### 4.4. Analysis

The first 100 ns of each simulation were regarded as further equilibration time and hence dismissed in these analyses. For all quantities analysed, we used the standard deviation from the mean of the three individual simulations runs per system (n=3) as error estimates. For the analyses of geometry parameters and interaction energies, we used cpptraj [51] from the AmberTools suite. Further data analysis was performed by custom-made Jupyter Notebooks using Python [52] with Numpy [53]. Plotting was conducted with Matplotlib [54].

Hydrogen bonds were defined based on geometric criteria, i.e., a donor–acceptor distance not larger than 3.2 Å and a donor–hydrogen–acceptor angle deviating from linearity by not more than 42°. This or similar geometric definitions are widely accepted in simulations of peptides and proteins in solution. Moreover, such geometric defintions have been justified by quantum chemically calculated interaction energies [55].

Molecular mechanical interaction energies between residues in the CPA–ligand complexes were calculated using the LIE command of Cpptraj [51] with default settings.

For determining the metal coordination geometry, we used the FindGeo tool [56] for all frames of the saved trajectories (i.e., every 100ps). The tool identifies metal ions and their coordinating atoms that are no farther than ~2.8Åaway. The coordination geometry is then compared to a library of various ideal geometries, and the geometry with the lowest root mean square deviation is considered as the best-fitting one.

Medoid structures (structures whose Cα positions deviate the least from the average structure of the respective run) for visualisation were obtained with Cpptraj [51] scripts. VMD 1.9.4 [57] was used to generate molecule figures.

## 5. Conclusions

We have modelled the Zn-dependent enzyme carboxypeptidase A complexed with short peptide ligands, in two different protonation states, and quasi-peptide ligands, each in D- and L-configuration. All ligands are modelled in a variant with either Tyr or Phe as the terminal residue. The Phe-containing quasi-peptides are known inhibitors with different efficacy. In all models, the ligands are rather well accommodated in CPA’s active site but with variations in their interactions with the protein residues and the coordination of the central Zn ion.

According to our simulations, the D-forms of the Phe-containing ligands have the weakest interactions and are least well positioned for hydrolysis. These data suggest that the strength of the inhibitory effect of the quasi-peptide ligands is not due to their affinity to bind to CPA, but rather due to the mode they bind to and interact with the active site of the enzyme. In particular, the Zn ion coordination seems to govern the mode of inhibition by hindering the activation and subsequent approach of a water molecule, whether in a direct attack through a “promoted water” mechanism or as a second step of an “acyl intermediate” mechanism.

Further studies will comprise extensive classical and quantum chemical simulations (such as simulations of ligand exchange at the zinc ion and reaction-path calculations) to obtain deeper insight into this somehow unusual mechanism of inhibition.

## Figures and Tables

**Table 1:** PDB structures to build the models, and abbreviations for the ligands used in this work. For the structures of the quasi-peptide ligands, see Figure 2.

N-Terminal Part	Ligand	Ligand	PDB
Charged glycine	GLY-TYR	GLY-PHE	3CPA
Neutral glycine	NGY-TYR	NGY-PHE	3CPA
(N-hydroxyamino)carbonyl	D-HAC-TYR	D-HAC-PHE	1HDQ
(N-hydroxyamino)carbonyl	L-HAC-TYR	L-HAC-PHE	1HEE
(amino)carbonyl	D-NAC-TYR	D-NAC-PHE	1HDU
(amino)carbonyl	L-NAC-TYR	L-NAC-PHE	1HDU

## Data Availability

Force field parameters of the neutral glycine residue and of the ligands as well as settings of the bonded models are available as Supplementary Material. Data series of quantities analysed and coordinates of the medoid structres are available as Supplementary Material.

## Supplementary Materials

The following supporting information can be downloaded at: https://www.mdpi.com/article/10.3390/ijms252413725/s1.

## Abbreviations

The following abbreviations are used in this manuscript:

CPA	Carboxypeptidase A
MD	Molecular Dynamics
HAC	(N-hydroxyamino)carbonyl
NAC	(amino)carbonyl
PDB	Protein Data Bank
