# Supplementary material for: Mode of Metal Ligation Governs Inhibition of Carboxypeptidase A
Source: Int J Mol Sci. 2024 Dec 23;25(24):13725. doi: 10.3390/ijms252413725 (PMC11677197; doi:10.3390/ijms252413725)
Supplement: Supplementary file 1 [file ijms-25-13725-s001.zip › CPA.SI.pdf]

Supplementary Information for - Mode of metal ligation governs inhibition of Carboxypeptidase A

Jorge Antonio Amador Balderas[1], Frank Beierlein[1,2], Anselm H. C. Horn[2,3], Senta Volkenandt[1], Leon Völcker[1], Nikoo Mokhtari[1], Jules Cesar Epee Ndongue[1], and Petra Imhof[1]*

December 5, 2024

[1] Department for Chemistry and Pharmacy
Computer Chemistry Center
Friedrich-Alexander University (FAU) Erlangen Nürnberg
Nägelsbachstraße 25
91052 Erlangen
Germany

[2]Erlangen National High Performance Computing Center (NHR@FAU)
Friedrich-Alexander University (FAU) Erlangen Nürnberg
Martensstraße 1
91058 Erlangen
Germany

[3]Institute of Biochemistry
Friedrich-Alexander University (FAU) Erlangen Nürnberg
Fahrstraße 17
91054 Erlangen
Germany

Fluctuations

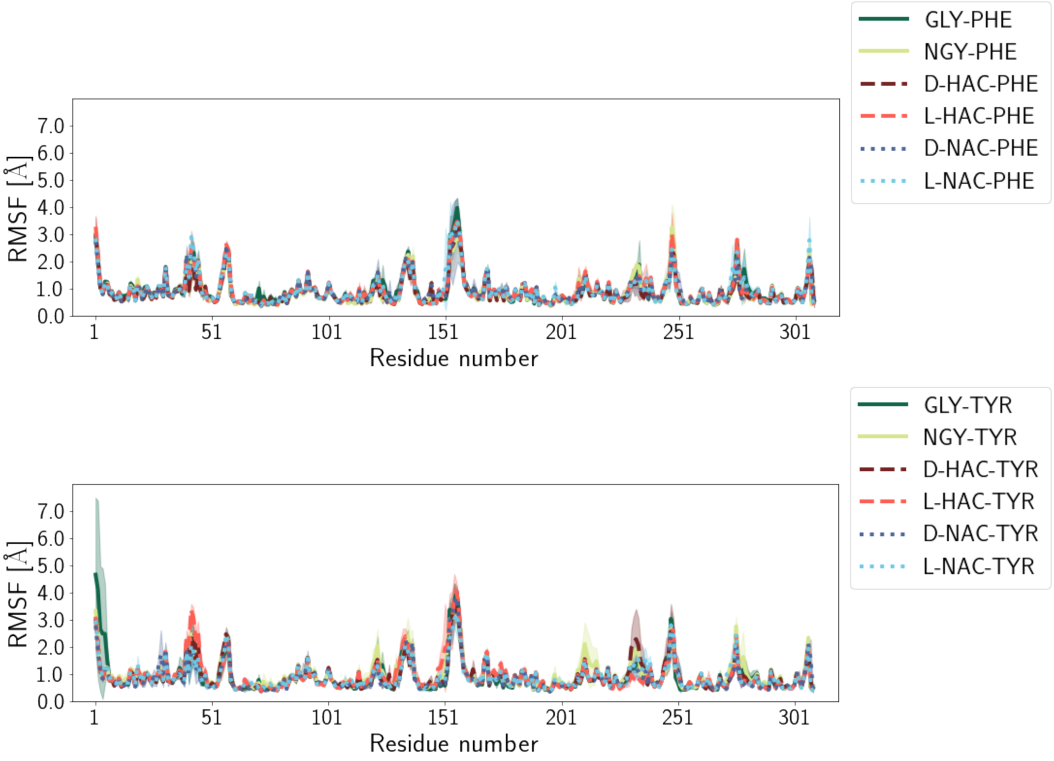

Figure S1: Fluctuations of the protein residues. Different colours refer to different ligands. Different ligands are shown in different colours and different N-terminal parts are indicated by different line styles.

Interaction Energies

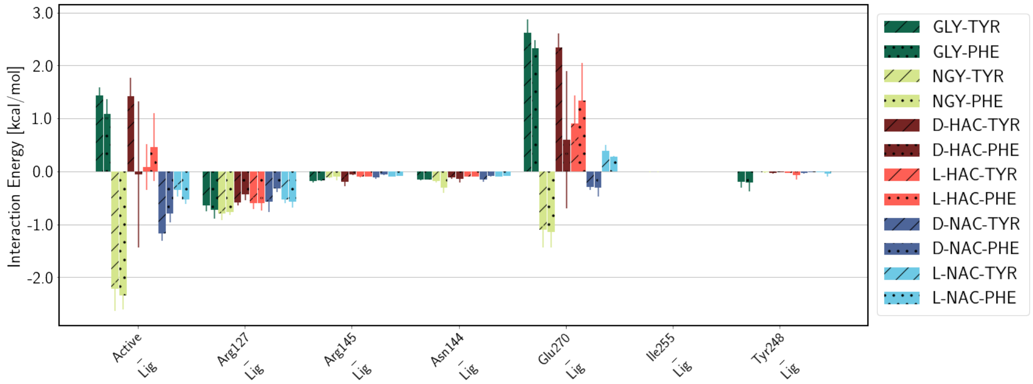

a)

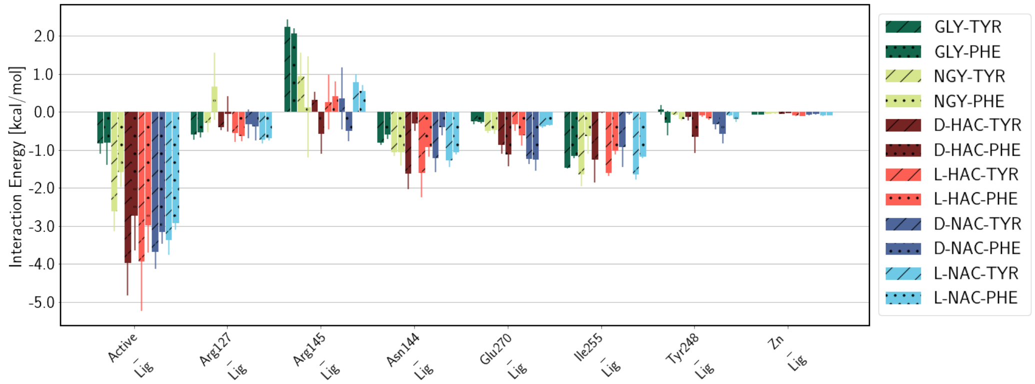

b)

Figure S2: VdW interactions between active-site residues and a) the N-terminal and b) the C-terminal part of the ligand. Different colours refer to different ligands (see legend). C-terminal tyrosine and phenylalanine are indicated by diagonal lines or dots, respectively.

Distances

According to the analysis of distances between active-site residues and the ligands, the carboxyl termini are farther from Arg145 and Asn144 in the ligands with D-Phe and in NGY-PHE, compared to the other models. GLY-TYR/PHE exhibit the shortest distances to Arg145. Distances to Arg127, in contrast are rather independent of the nature of the C-terminus or the N-terminus of the ligand. The shortest distances to Arg127 can be observed to the peptide ligands with neutral glycine, NGY-TYR and NGY-PHE, whereas all other ligands exhibit similar distances to this residue (see Figure S3). There is, however, some variability of the Arg127 and ligand atoms (NH1 or NH2 for Arg127) that are closest to each other.

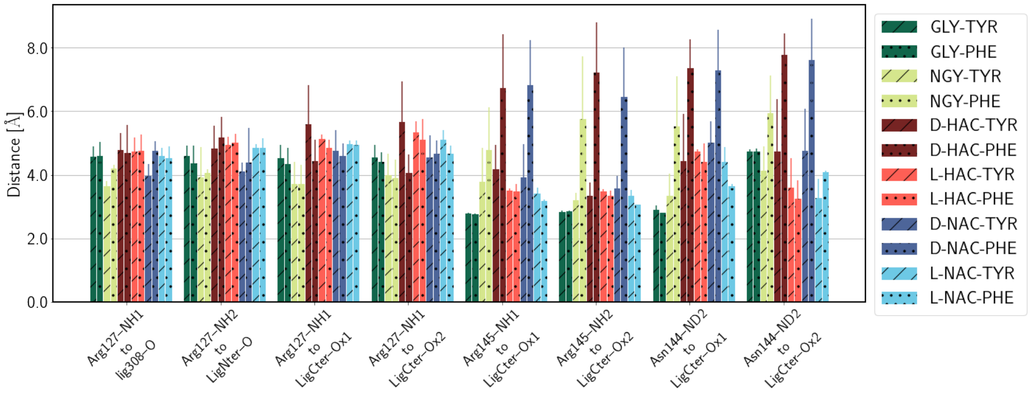

Figure S3: Distances between Arg or Asn active site residues and ligand. Different colours refer to different ligands (see legend). C-terminal tyrosine and phenylalanine are indicated by diagonal lines or dots, respectively.

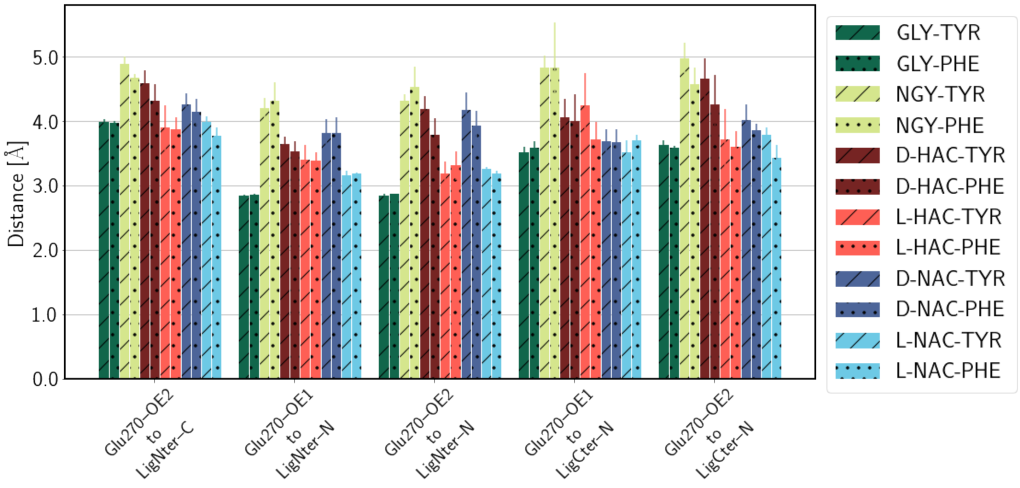

Figure S4: Distances between Glu270 and the ligands carbonyl C-atom and N-atoms (N- and C-terminal part). Different colours refer to different ligands (see legend). C-terminal tyrosine and phenylalanine are indicated by diagonal lines or dots, respectively.

Glu270 is closer to the N-terminal part of the ligands than to their C-terminal part, but not

by a large margin for NGY-TYR/PHE and for the D-form ligands D-HAC-TYR/PHE and D-NAC-TYR/PHE, as can be seen from the comparison of distances between this residue and the two N-atoms of the ligands (Figure S4). The N-termini of the GLY-TYR and GLY-PHE ligands are clearly within hydrogen bonding distance to Glu270 and the L-forms of the NAC and HAC ligands are also close enough to allow formation of hydrogen bonds (see also section on hydrogen bonds below). The D-forms of those ligands are somewhat farther and, most strikingly, the NGY-TYR/PHE ligands exhibit the longest distance to Glu270. This is in agreement with the short distance of the N-atom to the Zn-ion and underlining a different orientation of the N-terminal part of this NGY residue compared to the charged GLY residue in the other peptide ligands GLY-TYR/PHE.

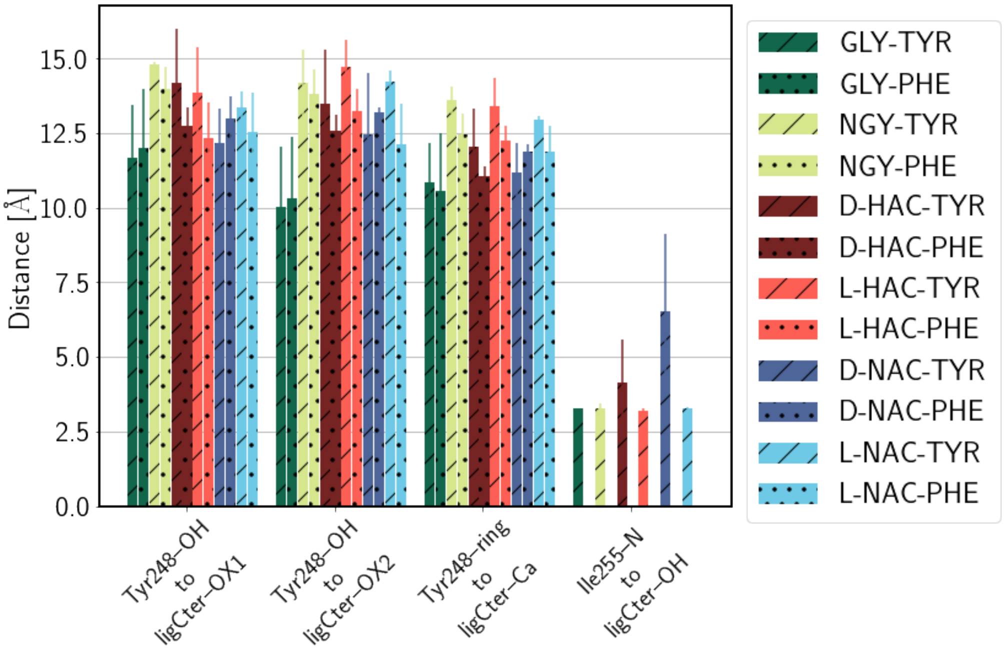

Figure S5: Distances between Tyr248 and the ligand's carboxyl O-atoms, OX1 and OX2, and Cα atom (Ca). Different colours refer to different ligands (see legend). C-terminal tyrosine and phenylalanine are indicated by diagonal lines or dots, respectively.

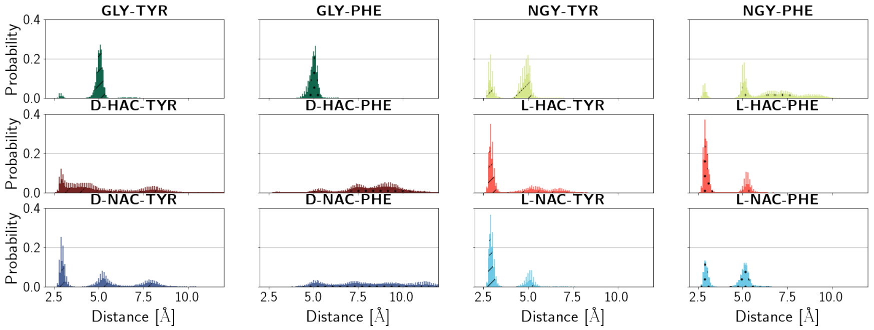

Figure S6: Distances between Asn144-ND2 and the closest C-terminal oxygen atom, OX1 or OX2, of the ligand. Different colours refer to different ligands (see legend). C-terminal tyrosine and phenylalanine are indicated by diagonal lines or dots, respectively.

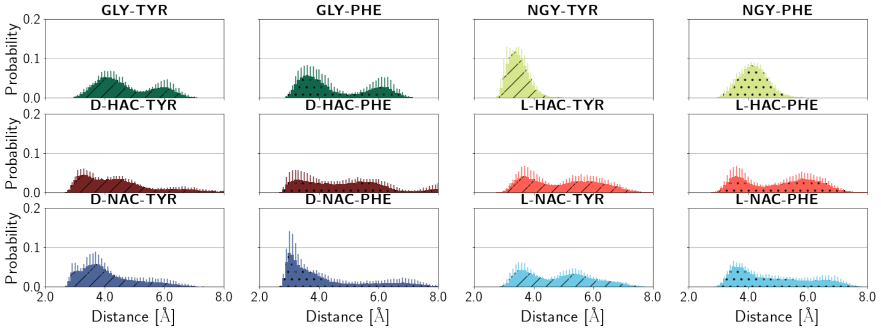

Figure S7: Distances between Arg127-ND and the closest C-terminal oxygen atom, OX1 or OX2, of the ligand. Different colours refer to different ligands (see legend). C-terminal tyrosine and phenylalanine are indicated by diagonal lines or dots, respectively.

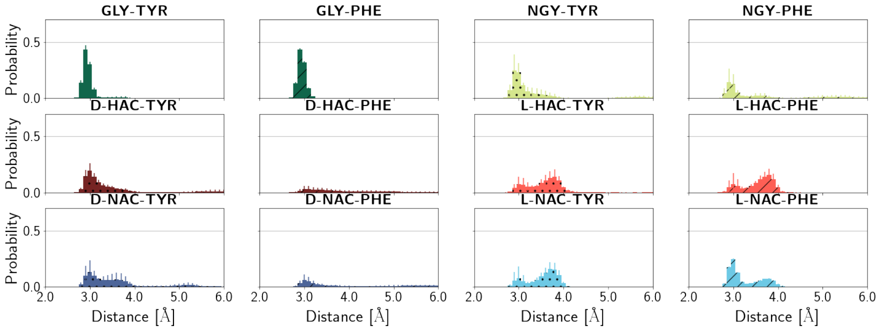

Figure S8: Distances between Arg145-ND and the closest C-terminal oxygen atom, OX1 or OX2, of the ligand. Different colours refer to different ligands (see legend). C-terminal tyrosine and phenylalanine are indicated by diagonal lines or dots, respectively.

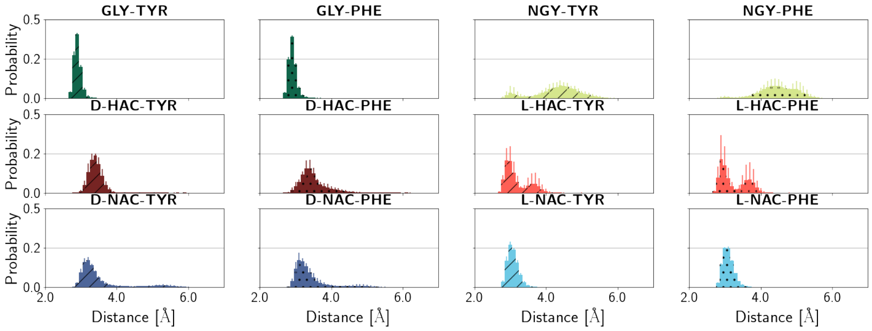

Figure S9: Closest distances between Glu270 carboxyl oxygen atoms, OE1 or OE2, and the ligand's carbonyl O-atom. Different colours refer to different ligands (see legend). C-terminal tyrosine and phenylalanine are indicated by diagonal lines or dots, respectively.

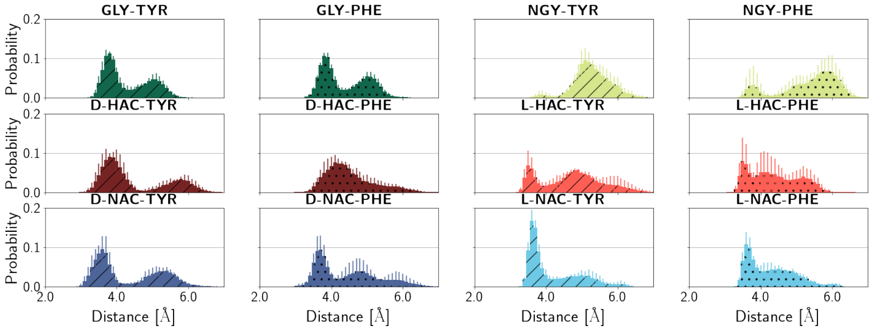

Figure S10: Closest distances between Glu270 carboxyl oxygen atoms, OE1 or OE2, and the ligand's carbonyl C-atom. Different colours refer to different ligands (see legend). C-terminal tyrosine and phenylalanine are indicated by diagonal lines or dots, respectively.

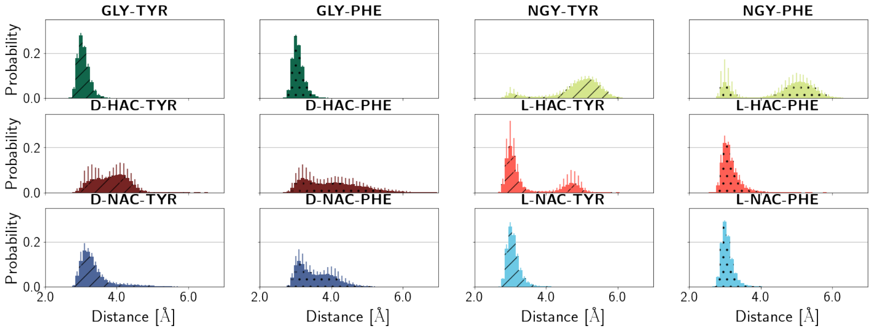

Figure S11: Closest distances between Glu270 carboxyl oxygen atoms, OE1 or OE2, and the ligand's amide N-atom. Different colours refer to different ligands (see legend). C-terminal tyrosine and phenylalanine are indicated by diagonal lines or dots, respectively.

Hydrogen Bonds

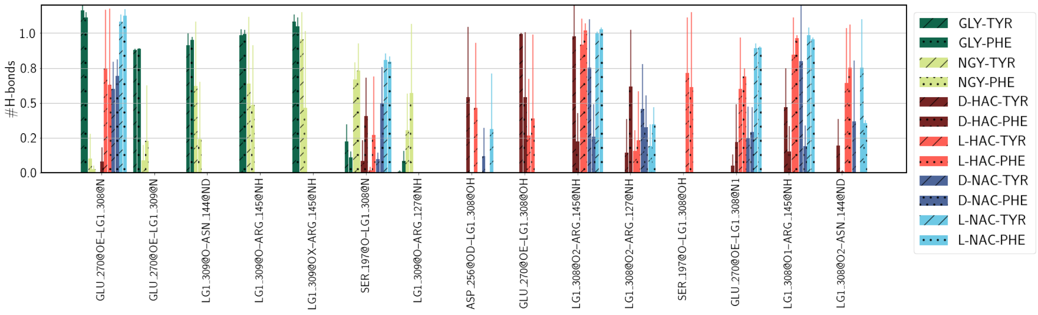

Figure S12: Hydrogen bonds between active-site residues and the ligand, with details on donor- and acceptor groups. Different colours refer to different ligands (see legend). C-terminal tyrosine and phenylalanine are indicated by diagonal lines or dots, respectively.

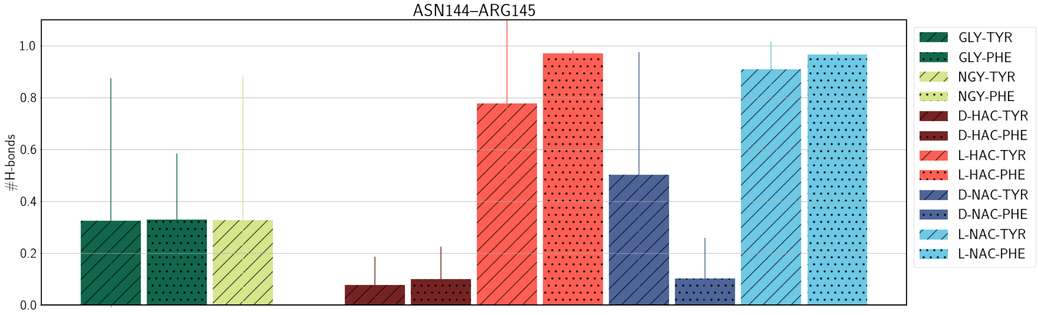

Figure S13: Hydrogen bonds between active-site residues. Different colours refer to different ligands (see legend). C-terminal tyrosine and phenylalanine are indicated by diagonal lines or dots, respectively.

Radial distribution functions

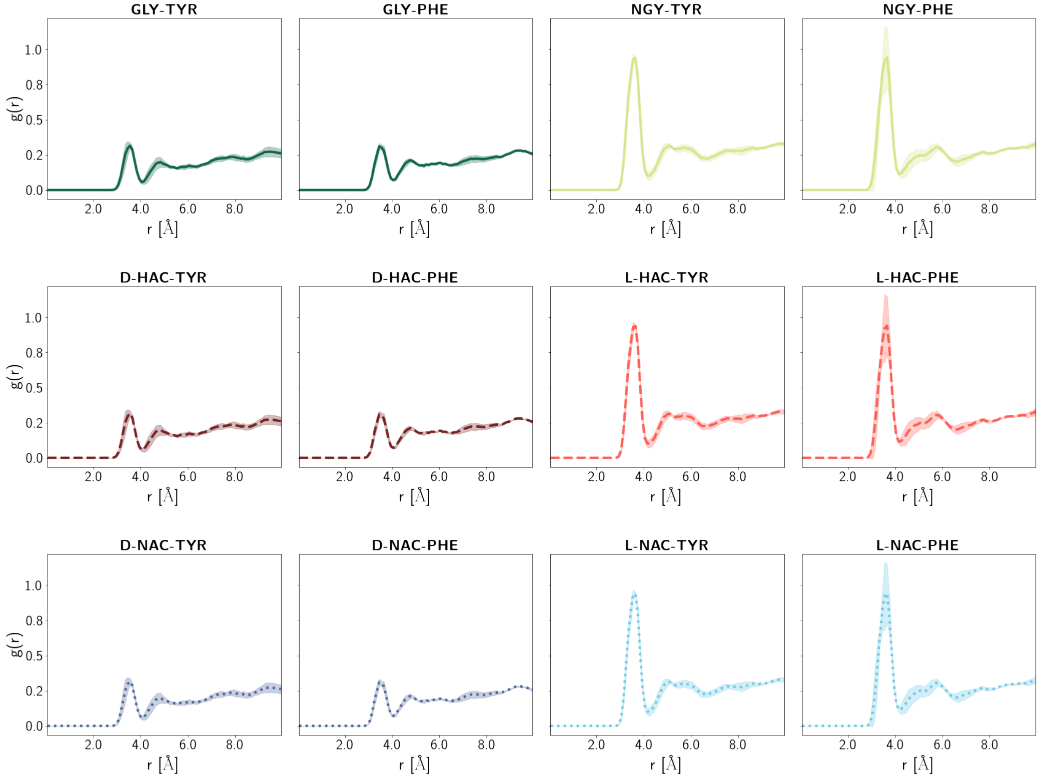

Figure S14: Radial distribution function of water molecules around the carboxyl C-atom of Glu270. Different colours refer to different ligands. Different N-terminal parts are indicated by different line styles.
